# On an Affordable Approach towards the Diagnosis and Care for Prostate Cancer Patients Using Urine, FTIR and Prediction Machines

**DOI:** 10.3390/diagnostics12092099

**Published:** 2022-08-30

**Authors:** Ejay Nsugbe, Hooi-Leng Ser, Huey-Fang Ong, Long Chiau Ming, Khang-Wen Goh, Bey-Hing Goh, Wai-Leng Lee

**Affiliations:** 1 Nsugbe Research Labs, Swindon SN1 3LG, UK; 2 Department of Biological Sciences, School of Medical and Life Sciences, Sunway University, Bandar Sunway 47500, Malaysia; 3 School of Information Technology, Monash University Malaysia, Bandar Sunway 47500, Malaysia; 4 PAPRSB Institute of Health Sciences, Universiti Brunei Darussalam, Gadong BE-1410, Brunei; 5 Faculty of Data Science and Information Technology, INTI International University, Nilai 71800, Malaysia; 6 Biofunctional Molecule Exploratory (BMEX) Research Group, School of Pharmacy, Monash University Malaysia, Subang Jaya 47500, Malaysia; 7 College of Pharmaceutical Sciences, Zhejiang University, 866 Yuhangtang Road, Hangzhou 310058, China; 8 School of Science, Monash University Malaysia, Subang Jaya 47500, Malaysia

**Keywords:** prostate cancer, FTIR, extracellular vesicles, LSDL, signal processing, oncology, machine learning, public health

## Abstract

Prostate cancer is a widespread form of cancer that affects patients globally and is challenging to diagnose, especially in its early stages. The common means of diagnosing cancer involve mostly invasive methods, such as the use of patient’s blood as well as digital biopsies, which are relatively expensive and require a considerable amount of expertise. Studies have shown that various cancer biomarkers can be present in urine samples from patients who have prostate cancers; this paper aimed to leverage this information and investigate this further by using urine samples from a group of patients alongside FTIR analysis for the prediction of prostate cancer. This investigation was carried out using three sets of data where all spectra were preprocessed with the linear series decomposition learner (LSDL) and post-processed using signal processing methods alongside a contrast across nine machine-learning models, the results of which showcased that the proposed modeling approach carries potential to be used for clinical prediction of prostate cancer. This would allow for a much more affordable and high-throughput means for active prediction and associated care for patients with prostate cancer. Further investigations on the prediction of cancer stage (i.e., early or late stage) were carried out, where high prediction accuracy was obtained across the various metrics that were investigated, further showing the promise and capability of urine sample analysis alongside the proposed and presented modeling approaches.

## 1. Introduction

In men, prostate cancer is the most frequently diagnosed cancer in 112 countries from North and South America, Australia, Europe and Africa, with a mortality rate of 7.7 per 100,000 worldwide [1]. There has been substantial debate in recent years regarding both the sensitivity and specificity of the prostate-specific antigen (PSA), particularly in the range of 4–10 ng/ml which is now known as the gray area in prostate cancer detection [2]. Men who tested with this range of the PSA level should seriously consider digital rectal examination (DRE) or biopsy to confirm the diagnosis. However, the invasiveness of these medical procedures is often the hurdle of timely cancer detection [3]. Liquid biopsy is a relatively simple and noninvasive alternative to biopsy. In comparison to blood samples, urine is easy to collect and could provide a range of information for the detection of cancers and other diseases. Therefore, urine-based liquid biopsy has been increasingly used for the diagnosis and follow-up of cancer patients [4]. Nevertheless, the vast biological variability found in complex liquid biopsy specimens may mask specific molecules and hinder the identification of cancer-specific changes. Extracellular vesicles (EVs), which are membranous nanoparticles secreted by living cells, can be found in all types of biofluids, including urine. Exosomes, a specific type of EVs formed as part of the endosomal pathway, have recently gained research interest due to their association with cancer progression [5]. Analysis focusing on these vesicles in biofluids could remove the abundant uninformative and uncorrelated data to effectively identify markers for cancer detection.

The use of computational methods within clinical medicine has seen a rapid rise in its applications over recent years, largely due to the availability of greater computational power and greater awareness of what these approaches can offer clinical settings in terms of improved disease diagnosis capability, while also allowing for an enhancement in care strategies that can be offered to patients [6,7]. In particular, machine-learning methods have been deeply researched in the area of cancer for such tasks as prediction of the presence of cancerous cells, survival analysis, and even treatment scheduling and optimal dosing of treatment therapies for patients [8,9]. With the emphasis on prostate cancer, machine-learning methods have been employed mostly towards the binary prediction of the presence of cancers, where the primary sources of data include pathological samples, radiological instrumentation, and blood samples [10,11,12,13,14,15].

Recently, it has been observed that urine carries relevant biomarker antigens which, when analyzed using the appropriate spectroscopic tools alongside the necessary signal processing, can allow for the reliable prediction of the presence of prostate cancer [16,17,18,19,20]. This carries a high appeal due to the acquisition of urinary samples being a noninvasive process with a high-throughput biomarker analysis and thereby allows for a cheap and noninvasive means towards the diagnosis of prostate cancer [16,17,18,19,20]. It has also been shown that Fourier-transform infrared (FTIR) spectroscopy can be employed as part of the sample analysis process [21,22].

The linear series decomposition learner (LSDL) is an artificial intelligence (AI) approach formed in the area of metaheuristics, that is capable of performing a sequence of decompositions on a candidate signal with the use of a linear basis function with the goal of performing signal separation in order to obtain the optimal region of the signal which minimizes uncertainty and redundancy within the signal and maximizes the information quality within the candidate signal [23,24,25,26,27]. The LSDL has seen applications beyond its inception case study, which was based around source separation of an acoustic emission signal, and has seen use in the processing of various other time series in clinical areas spanning rehabilitation, pregnancy and psychiatric medicine, where the inclusion of the LSDL as a signal decomposition tool prior to modeling and prediction exercises was noted to boost the overall prediction accuracy within each case study [28,29,30,31,32,33]. An expanded description of the architecture of the LSDL can be seen in Section 2.2.

Recently, the LSDL has been trialed on spectroscopic waveforms—which can be noted to differ from a typical time series—where FTIR spectra were obtained from blood samples for the diagnosis of endometrial cancer [34]. The pilot work involving the implementation of the LSDL showed once again an increment in the model predictive performance upon decomposition with the LSDL beforehand, thereby also showing the potential of the LSDL in the processing of spectroscopic waveforms, hence paving the way for further research exploration.

Thus, in this paper, given an acquired urine sample which is subsequently analyzed with FTIR instrumentation to obtain its respective spectrum, the application of the LSDL to the decomposition of urine spectroscopy signals for enhanced and improved prediction of the presence of prostate cancer is observed for the first time. Specifically, the contributions of this paper are as follows:

1. Application of the LSDL towards decomposition of the obtained spectra from FTIR urine from a patient population in order to postprocess and build prediction models that can predict the presence of prostate cancer using urine samplefs.
2. Comparison of the results and predictive performance of the LSDL and raw spectroscopy across nine different machine-learning models.
3. Further prediction exercises around the stage of prostate cancer for early- and late-grade variants of the disease.

### 1.1. Urine Biomarkers as a Means towards Prostate Cancer Diagnosis

In the clinical screening of prostate cancer, the use of the prostate-specific antigen (PSA) is the predominant means towards the diagnosis of prostate cancer, although this approach is limited due to the potential of the PSA levels to also be elevated in cases where there is benign prostate hyperplasia and prostatic inflammation [16,17,18,19]. Even such activities as cycling and sex can raise the PSA levels.

**Figure 1:**
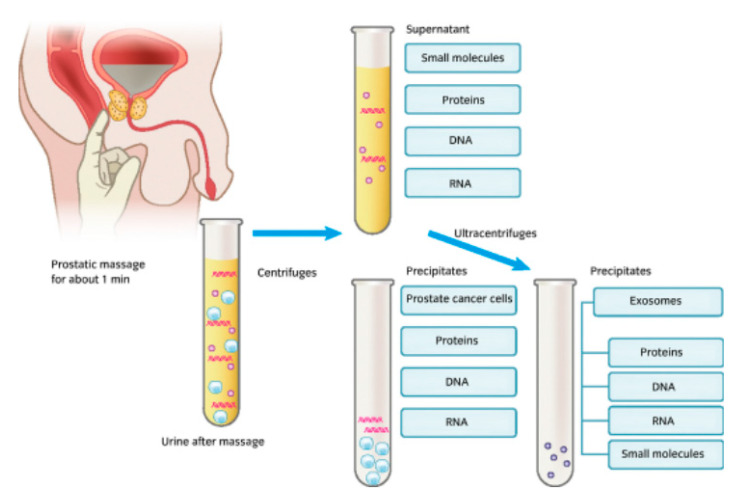
An image showing a form of urine collection involving prostate stimulation beforehand, the biomarker constituents within urine, and the laboratory-based constituent separation process [35].

It is known that after the manipulation of the prostate, prostatic fluids from prostate cancer cells are released alongside urine, and in certain cases these fluids can also be released even without urinary manipulation [35]. Here, the chief candidate biomarkers released as part of the urinary process included DNA, RNA, and proteins, as well as a host of other small molecules, as seen in Figure 1.

### 1.2. IR Data Acquisitions

In the previous pilot study [36], urinary EV samples were collected from 53 treatment-naïve patients suspected of having prostate cancer. Each of these individuals underwent transrectal ultrasound (TRUS)-guided prostate biopsy at the University of Malaya Medical Centre between 2016 and 2019. These specimens were divided into two groups of 31 noncancerous and 22 cancerous specimens according to the biopsy outcome. Further, 22 cancerous samples were grouped into three Gleason score (GS) groups: four samples with GS < 7, five samples with GS = 7 and 13 samples with GS > 7. All the urinary EV samples were analyzed using attenuated total reflection–Fourier-transform infrared spectroscopy (ATR–FTIR). A total of 53 spectra acquired from the pilot study [36] were used in the following data analysis.

## 2. Materials and Methods

### 2.1. Datasets

#### 2.1.1. Cancer vs. No Cancer

Dataset 1: this dataset involved the use of samples which were acquired using a repetition format where three iterations were taken from the urine sample and averaged out in order to form the final spectroscopic readings that were eventually used. With the aid of the SMOTE synthetic sample generator, the number of data samples was increased to a total of 62 samples, of which 31 belonged to cancer patients and the remaining 31 were from noncancer patients in a balanced dataset.

Dataset 2: for this dataset, the same patients’ urine samples were used, but this time, no repetition or averaging process was conducted as only a single acquisition from each urine sample was used to create the final FTIR readings. Similarly, to Dataset 1, a sum of 62 samples was obtained for this group with a balance in both classes, driven by the use of the SMOTE algorithm.

Dataset 3: for this dataset group, concatenation of the data from Datasets 1 and 2 was performed, which meant that this group comprised the FTIR samples acquired using the averaging method and also the ones acquired using the single-acquisition approach, resulting in a total of 124 samples, which meant that this dataset was the largest group.

The cumulative number of sample points used as part of this exercise was 248 across all three data groups.

#### 2.1.2. Low- vs. High-Grade Cancer

For this exercise, only the dataset of patients who had cancer was used. Patients who had prostate cancer scores of 7 were designated to the class of low-grade cancer, while patients whom had prostate cancer scores in the range of 8–10 were designated to the class of a high-grade cancer.

Dataset 1: this dataset was comprised of FTIR data which were averaged from three different acquisitions, as previously mentioned, using the SMOTE algorithm for class balancing purposes. The sum of samples in this group was 40, with a balanced group of samples within each data class.

Dataset 2: this group of data was formed using single acquisitions from the urine samples to acquire the FTIR readings, and also comprised of a class of 40 samples, which were balanced using the SMOTE algorithm.

Dataset 3: this dataset was comprised of a concatenation of the data from the prior groups to form a total of 80 samples of data from both the averaged and single sampled approach.

For this case, the cumulative number of 160 samples was utilized for the analysis carried out for this exercise.

### 2.2. Signal Decomposition and LSDL

The LSDL, as mentioned, is an AI-driven method towards the signal deconvolution process built upon metaheuristic reasoning, with the original application of the approach based around source separation of a high-frequency acoustic emission signal in order to infer the particle size distribution of a heterogenous powder mixture [28,29,30,31,32,33]. Its performance has been observed to surpass that of the wavelet transform in terms of accuracy and computational efficiency [28,29,30,31,32,33].

In the case of events which occur simultaneously, the resulting time domain signal contains an overlapping impulse, with magnitude (also referred to as amplitude in this case) being a key characteristic towards effective characterization of the source events, as described by Nsugbe et al. [37,38,39,40]. This gives rise to the hypothesis that an optimal amplitude region within a candidate signal is the one whose information quality is maximized as adjudged by a designated performance index and, thus, contains minimal uncertainties [27].

Following its original application involving source separation and estimation of particle size distribution, the LSDL has seen further applications in case studies that involve brain–machine interface control of bionic upper limb prostheses, prediction of preterm pregnancies from uterine contraction signals, prediction of adolescent schizophrenia from EEG brainwave signals and, more recently, prediction of depth of anesthesia during surgery using frontal cortex neural oscillations [27,31,32,33,37,38].

The LSDL is based around the separation of a given signal into various amplitude bands by way of selection of tuned linear thresholds, which serve as the basis function for the decomposition problem, alongside a peak detection algorithm which identifies characteristics of a given signal as a “peak” of sorts within a tuned threshold level. The term “LSDL” represents the fact that a linear basis function is used for the signal decomposition process to yield a series of sub-signals, from which an optimal signal and the associated decomposition level are obtained and evaluated via an embedded learning process within the algorithm.

All decomposed regions from the tuned thresholds are subjected to an evaluation by a performance index to evaluate the information quality and discriminatory power of a particular signal threshold region with respect to a performance index of sorts. The performance index utilized as part of the LSDL is the normalized Euclidean distance which, given two sets of samples from two different classes, estimates the separation between them in Euclidean space. The mathematical framework for the normalized version of the Euclidean distance used is covered by Equations (1)–(3) [31]:

(1) $$ED\left(p,q\right)=\sqrt{{\left(p_{1}-q_{1}\right)}^{2}+{\left(p_{2}-q_{2}\right)}^{2}}$$

(2) $$\sigma =\sqrt{\frac{\sum_{w=1}^{N_{w}}{\left(r_{w}-\mu \right)}^{2}}{N_{w}}}$$

(3) $$J=\left(p,q\right)=\frac{ED\left(p,q\right)}{\sigma_{m}}$$

where ED is the Euclidean distance given coordinates *p* and *q*, *w* is the *w*th feature in feature vector $N_{w}$, $r_{w}$ is a specific feature in a feature vector, μ represents the mean of the features and $\sigma_{m}$ is the mean of the standard deviations of the features being considered.

**Figure 2:**
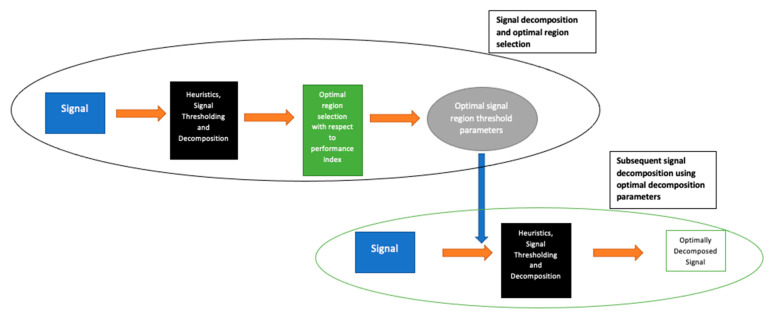
Flow diagram showing the various stages associated with the implementation of the LSDL.

As described, the first stage of the LSDL involves the iterative sorting and assessment of the decomposed region of the signal in order to find the optimal region of the signal which maximizes information quality. Once this is determined, all other subsequent signals from the same source are then decomposed with parameters which belong to the optimal signal region. An illustration of this is shown in Figure 2.

It can be mentioned that in addition to greater prediction accuracy, some of the benefits of the LSDL include the demand for less storage space due to its decomposition act and high computational efficiency, where its computational complexity is observed to be in the order of O(n).

#### Threshold Selection Exercise

For the calculation of the optimal threshold region with the LSDL, the Cancer vs. No Cancer datasets were used, obtained using the implementation parameters from Nsugbe et al. [31] and Nsugbe and Sanusi [32]. The results can be seen in Table 1 and Table 2.

According to the results in Table 1 and Table 2, although the optimal region is in the first iteration of the upper threshold region, the values appear to differ, which is indicative of the notion that different sample processing methods have the potential to impact results.

Note that decomposition parameters were not computed for Dataset 3 as this dataset group comprised a concatenation of Datasets 1 and 2.

### 2.3. Feature Extraction

The following features were extracted from the spectroscopy signals, as had been applied in previous studies [39,40,41]: mean absolute value (MAV), waveform length (WL), slope sign change (SSC), fourth autoregressive coefficient (4thAR), sample entropy (SampEN), cepstral coefficient (Ceps), maximum fractal length (MFL), Higuchi fractal dimension (HFD), detrended fluctuation analysis (DFA), median frequency (MF) and sum of peaks (SP). For the features which require a threshold, a value of 1 µv was used, while for the number of signal peaks, a peak could be defined as $\{\begin{array}{c} x_{peak.n},x_{n}\geq x_{n-1}\;and\;x_{n+1}\\ 0,\;Otherwise \end{array}$.

It should be noted that due to the nature of the LSDL algorithm, the ZC, DFA and MF features were seen to be redundant.

### 2.4. Machine Learning

Decision tree (DT): a nonparametric model which works with a Boolean-like approach towards a systematic classification of samples into various data classes in a hierarchical tree-like flow, also viewed as a nonparametric approach [42].

Discriminant analysis: classification model based around statistical reasoning, works with the projection of what would ordinarily be a high-dimensional data vector into a lower-dimensional projection, followed by the imposition of class boundaries [43]. Linear and quadratic variants of discriminant analysis were used in this paper, i.e., LDA and QDA.

K-nearest neighbors (KNN): model utilizing a voting strategy based on the nearest neighbor to designate samples into respective data classes [44]. Here, the Euclidean distance metric was utilized as the distance metric of choice while K was chosen to be 1.

Support vector machine: an iterative classification method based around the computation of the optimal decision boundary that maximizes the separation between data classes while using a subset of the data referred to as support vectors [45]. The instillation of class boundaries is performed in a higher-dimensional space before being subsequently downscaled whilst the overall structure is preserved in a process referred to as the kernel trick. Linear, quadratic, cubic and fine gaussian kernels were used in this paper, hence LSVM, QSVM, CSVM and FGSVM.

Logistic regression (LR): A statistically driven method which applies the sigmoid activation function alongside an accompanying threshold towards the classification of data from different classes in a binary-like fashion [46].

All classification models were validated using a K-fold cross-validation method where K was chosen as 10.

All model training and development were performed with the MATLAB classification learner application which tunes for the optimal hyperparameter for each selected model given the criteria selected.

## 3. Results and Discussion

For the quantification of the results, the following metrics were employed: classification accuracy (Acc; provides a metric to quantify the amount of samples correctly classified), sensitivity (Sens; allows for the quantification of the samples which were correctly classified and were actually positive as a ratio of the total samples within the class), specificity (Spec; provides a measure of the amount of samples which were correctly classified as negative expressed as a ratio of the total samples in the class), and area under the curve (AUC; provides a metric which can be used to assess the model’s overall performance by taking into account the sensitivity and specificity, as has been used previously for other related studies) [27].

### 3.1. Cancer vs. No Cancer

#### 3.1.1. Dataset 1

The results for Dataset 1 for this exercise can be seen in Table 3 and Table 4 for the raw data and LSDL decomposed, respectively. In terms of the raw data, the best performance was observed to be from QDA, followed by the DT, implying that discriminant analysis with a nonlinear/quadratic decision boundary as well as the DT (which works off a Boolean logic function) are optimal for datasets of these kinds.

In terms of the LSDL results for Dataset 1, a considerable increase in the overall predictive performance could be observed for all the models, where the best performing model was LDA alongside LR. This showcases the impact of the LSDL on the overall model performance, with a strongly improved predictive prowess all round, which highlights the effect of the algorithm on enhancing the prediction capability of the detection of prostate cancer using urine samples.

#### 3.1.2. Dataset 2

In terms of Dataset 2, which represents the data acquisition process where repetitions were utilized, the results showed an overall drop in the model’s predictive performance with exceptions of KNN, CSVM and FGSVM, as seen in Table 5. This implies that, for the raw data exercise, these kinds of models are best suited to cases where the repetition FTIR acquisition method is adopted. Although it is unclear which means towards data acquisition produce the most accurate representation of the patient’s urine sample, the law of ergodicity implies that a repeated number of sample repetitions produces a closer approximation towards the real phenomena being investigated and, as per the obtained results, there was a notable amount of variation between the prediction accuracy and model performance between the data acquired with and without any form of repetition.

As in the case of the raw data, the results of the LSDL reflected the same findings in the sense that there appeared to be an overall drop in model performance across the majority of the classifiers considered, again reflecting the degradation in performance when the repetition-based acquisition method is used, although it can be seen that the DT and LSVM’s performances appeared to be marginally improved. Overall, however, the LSDL continued to show superior model’s predictive performance when compared with the raw data despite the mode of data acquisition, as seen in Table 6.

#### 3.1.3. Dataset 3

The case of Dataset 3 showcased a mixture of model-based performance, with the performance of certain models improving while others degraded slightly, as seen in Table 7. This dataset can perhaps be deemed to be a truer representation of the actual model behavior as it contained the largest sample size, with a reasonable amount of variability stemming from the different modes of data acquisition. Here, it can be seen that the best predictive performances were from the DT and LR models.

The same trend could be observed for the LSDL in this case as well, where it can be seen in Table 8 that the best model predictors belonged to the CSVM, QSVM and QDA, showing that nonlinear decision boundaries appeared to be optimal in this case.

### 3.2. Cancer Extent

On the basis of a positive cancer prediction, this section looked at a trained model’s ability to predict the associated stage of a cancer and serves as a useful source of auxiliary information in the prioritization of patient care, etc.

For the prediction exercises carried out in this section, the Gleason scores were chinked into two classes, i.e., scores of less than 7 and equal to 7 were grouped into one class (total of nine samples) while the Gleason scores over 7 were taken to form a separate class comprising 13 samples. As per the previous section, the SMOTE algorithm was utilized towards increasing the number of samples as well as class balancing.

As the best results in the prior sections were obtained for the LSDL, the subsequent prediction exercises in this section were performing using the LSDL decomposition as opposed to the raw data.

#### 3.2.1. Dataset 1

The results for Dataset 1 can be seen in Table 9, where it can be noted that, generally, high-prediction metrics are obtained for the various models, with LDA and LR providing the best predictive performance. This showcases that, under the investigated circumstances, it is possible to predict the extent and stage of cancer using the proposed method.

#### 3.2.2. Dataset 2

For this case, although the best model performance still belonged to LDA, it can be noted in Table 10 that the overall classifier model behavior improved across the various other models, implying that for the cancer extent modeling exercise, this means of data acquisition may provide the best results, in contrast to what was seen in the prior Cancer vs. No Cancer exercise.

#### 3.2.3. Dataset 3

The results for Dataset 3, which, as mentioned, represented a realistic group of data with a mixture of variabilities, can be seen in Table 11. It can be seen that there is a range of model’s predictive performances which boast a generally high accuracy across the various performance metrics, with LR providing the best predictive performance. The slight reduction in predictive performance of Dataset 3 when compared with prior datasets could be attributed to the broader sample size and variability posed as part of the Dataset as described, which has resulted in a slight reduction in the performance of the models.

**Figure 3:**
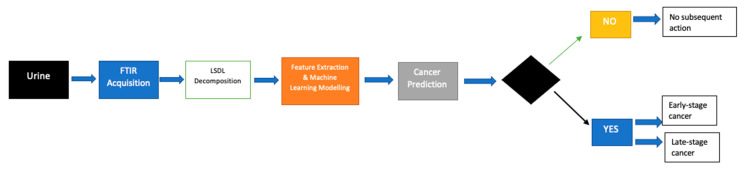
Flow diagram showing the operational flow of the proposed modeling approach.

**Figure 4:**
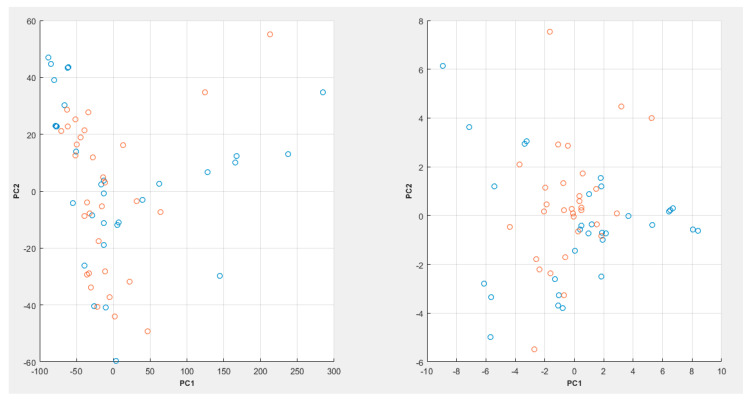
PCA plot for the raw spectroscopic data (**left**) and the LSDL decomposed data (**right**) with 98% variability explained (where blue = cancer samples, red = noncancer samples).

A full flow of the proposed process can be seen in Figure 3, while a PCA plot can be seen in Figure 4. 

In Figure 4, it can be seen that the PCA plot with the raw spectroscopic data appears to show a bit more cluster overlap between the various data classes, whereas in the case of the LSDL decomposed spectral data, greater cluster separability can be observed, which makes it easier for the classes to be separated and learned by a machine-learning model.

## 4. Conclusions

In this work, we conducted a pilot study based around the feasibility of using urinary EVs alongside FTIR analysis data for the prediction of prostate cancer and the subsequent inference of the extent of cancer due to the apparent presence of key cancer biomarkers within urinary EVs. Using three sets of data representing different variants of sample processing and acquisition with FTIR instrumentation, this work investigated the application of the LSDL decomposition method towards the preprocessing of FTIR spectra prior to machine-learning modeling. The results showed an improved predictive performance when benchmarked with the raw spectroscopic data for the three dataset variants, providing quantitative evidence that EVs isolated from urine samples can serve as an affordable means towards early and noninvasive detection of prostate cancer. Additional analysis also showed that using the presented method, it is possible to predict the associated stage of cancer where a positive cancer status has been identified. In terms of the model acceptability criteria, this is envisaged to differ between various clinics and hospitals but an arbitrary threshold cutoff of 70% could be viewed as proof of acceptable model performance.

The key limitations of this work revolve around the learning and tuning process required as part of the implementation of the LSDL which, due to the nature of the algorithm, is iterative, in addition to the sample size used as part of this pilot, which is believed to comprise a homogenous ethnic population. Further work in this area would involve the collection of data from a larger pool of patients, hopefully of various ethnicities, which would provide further reflective data for the approach to be validated against [47].

## Figures and Tables

**Table 1:** LSDL decomposition results for Dataset 1.

Threshold Level	Iteration 1	Iteration 2	Iteration 3	Iteration 4
Upper	2.5852 *	n/a **	n/a	n/a
Lower	2.0031	2.0028	2.0063	2.0259

* The threshold values were modified and varied slightly in order to compute the performance index for that particular threshold. ** Indicates an inadequate amount of signal samples for the computation of the performance index value.

**Table 2:** LSDL decomposition results for Dataset 2.

Threshold Level	Iteration 1	Iteration 2	Iteration 3	Iteration 4
Upper	2.0954 *	n/a **	n/a	n/a
Lower	2.0021	2.0043	2.0099	2.0288

* The threshold values were modified and varied slightly in order to compute the performance index for that particular threshold. ** Indicates an inadequate amount of signal samples for the computation of the performance index value.

**Table 3:** Results for the raw data for Dataset 1.

	Acc (%)	Sens (%)	Spec (%)	AUC (%)
DT	69	67	71	69
LDA	59	59	59	59
KNN	45	45	45	45
LSVM	54	57	53	55
QSVM	67	68	67	68
CSVM	48	48	48	48
FGSVM	50	50	50	50
LR	61	61	61	61
QDA	72	79	68	74

**Table 4:** Results for the LSDL for Dataset 1.

	Acc (%)	Sens (%)	Spec (%)	AUC (%)
DT	79	76	82	79
LDA	98	97	100	99
KNN	86	84	87	86
LSVM	86	84	87	86
QSVM	95	94	97	96
CSVM	94	91	97	94
FGSVM	79	91	73	82
LR	94	91	97	94
QDA	92	96	88	92

**Table 5:** Results for the raw data for Dataset 2.

	Acc (%)	Sens (%)	Spec (%)	AUC (%)
DT	48	48	48	48
LDA	53	53	53	53
KNN	63	62	63	63
LSVM	45	46	45	46
QSVM	61	60	62	61
CSVM	56	56	56	56
FGSVM	67	72	64	68
LR	50	50	50	50
QDA	55	55	54	55

**Table 6:** Results for the LSDL for Dataset 2.

	Acc (%)	Sens (%)	Spec (%)	AUC (%)
DT	86	87	84	86
LDA	90	93	88	89
KNN	86	84	87	86
LSVM	90	88	93	91
QSVM	90	86	96	91
CSVM	90	86	96	91
FGSVM	76	86	70	78
LR	94	94	94	94
QDA	90	93	88	91

**Table 7:** Results for the raw data for Dataset 3.

	Acc (%)	Sens (%)	Spec (%)	AUC (%)
DT	69	69	68	69
LDA	66	65	67	66
KNN	59	59	58	59
LSVM	52	53	52	53
QSVM	63	64	61	63
CSVM	55	54	56	55
FGSVM	58	61	56	59
LR	64	64	76	70
QDA	56	56	56	55

**Table 8:** Results for the LSDL for Dataset 3.

	Acc (%)	Sens (%)	Spec (%)	AUC (%)
DT	75	72	78	75
LDA	66	64	69	67
KNN	83	81	86	84
LSVM	58	57	73	65
QSVM	85	80	91	86
CSVM	86	83	88	86
FGSVM	78	89	72	81
LR	65	64	66	65
QDA	85	87	77	82

**Table 9:** Results for the LSDL for Dataset 1.

	Acc (%)	Sens (%)	Spec (%)	AUC (%)
DT	65	67	64	66
LDA	93	90	95	93
KNN	78	79	76	78
LSVM	78	79	76	78
QSVM	73	71	74	73
CSVM	75	78	73	76
FGSVM	80	77	83	80
LR	88	86	89	88
QDA	n/a	n/a	n/a	n/a

**Table 10:** Results for the LSDL for Dataset 2.

	Acc (%)	Sens (%)	Spec (%)	AUC (%)
DT	80	77	83	80
LDA	93	87	100	94
KNN	93	90	95	93
LSVM	73	68	80	74
QSVM	93	90	95	93
CSVM	90	90	90	90
FGSVM	85	94	79	87
LR	95	91	100	96
QDA	n/a	n/a	n/a	n/a

**Table 11:** Results for the LSDL for Dataset 3.

	Acc (%)	Sens (%)	Spec (%)	AUC (%)
DT	71	69	74	72
LDA	88	84	92	88
KNN	89	90	88	89
LSVM	74	71	77	74
QSVM	88	83	94	89
CSVM	86	89	84	87
FGSVM	78	81	75	78
LR	90	88	92	90
QDA	89	90	88	89

## Data Availability

The data are available upon reasonable request from the authors.
